# Unexpected cardiorespiratory findings postictally and at rest weeks prior to SUDEP

**DOI:** 10.3389/fneur.2023.1129395

**Published:** 2023-03-24

**Authors:** Yassine Lamrani, Thi Phuoc Yen Tran, Dènahin Hinnoutondji Toffa, Manon Robert, Arline-Aude Bérubé, Dang Khoa Nguyen, Elie Bou Assi

**Affiliations:** ^1^University of Montreal Hospital Research Center (CRCHUM), Montreal, QC, Canada; ^2^Department of Neuroscience, University of Montreal, Montreal, QC, Canada; ^3^Division of Neurology, University of Montreal Hospital Center (CHUM), Montreal, QC, Canada

**Keywords:** epilepsy, SUDEP, focal seizures, HRV, autonomic changes

## Abstract

**Introduction:**

Mechanisms underlying sudden unexpected death in epilepsy (SUDEP) are unclear, but autonomic disorders are thought to play a critical role. However, those dysfunctions have mainly been reported in the peri-ictal context of generalized tonic–clonic seizures. Here, we explored whether heart rate variability (HRV), heart rate (HR), and breathing rate (BR) changes could be observed perictally during focal seizures with or without impaired awareness as well as interictally to assess the risk of SUDEP. We report the case of a 33-year-old patient with drug-resistant bilateral temporal lobe epilepsy who died at home probably from an unwitnessed nocturnal seizure (“probable SUDEP”).

**Methods:**

Ictal and interictal HRV as well as postictal cardiorespiratory analyses were conducted to assess autonomic functions and overall SUDEP risk. The SUDEP patient was compared to two living male patients from our local database matched for age, sex, and location of the epileptic focus.

**Results:**

Interictal HRV analysis showed that all sleep HRV parameters and most awake HRV parameters of the SUDEP patient were significantly lower than those of our two control subjects with bitemporal lobe epilepsy without SUDEP (p < 0.01). In two focal with impaired awareness seizures (FIAS) of the SUDEP patient, increased postictal mean HR and reduced preictal mean high frequency signals (HF), known markers of increased seizure severity in convulsive seizures, were seen postictally. Furthermore, important autonomic instability and hypersensitivity were seen through fluctuations in LF/HF ratio following two seizures of the SUDEP patient, with a rapid transition between sympathetic and parasympathetic activity. In addition, a combination of severe hypopnea (202 s) and bradycardia (10 s), illustrating autonomic dysfunction, was found after one of the SUDEP patient’s FIAS.

**Discussion:**

The unusual cardiorespiratory and HRV patterns found in this case indicated autonomic abnormalities that were possibly predictive of an increased risk of SUDEP. It will be interesting to perform similar analyses in other SUDEP cases to see whether our findings are anecdotal or instead suggestive of reliable biomarkers of high SUDEP risk in focal epilepsy, in particular focal with or without impaired awareness seizures.

## 1. Introduction

Sudden unexpected death in epilepsy (SUDEP) refers to the sudden, unexpected, witnessed or unwitnessed, nontraumatic and nondrowning death in patients with epilepsy (1). SUDEP is not only the leading epilepsy-related cause of death (2), but also the second neurological cause of total potential life-years lost, only behind stroke (3). Mechanisms underlying SUDEP are still not fully understood but cardiorespiratory and autonomic dysfunctions in the postictal phase are generally thought to play an important role (4). Identified risk factors for SUDEP include generalized tonic–clonic seizures (GTCS), temporal lobe epilepsy, nocturnal seizures, and prone sleeping position (4). A history of GTCS and presence of GTCS in the last year were associated with a 10- and 27-fold increase in SUDEP risk, respectively (5). In recent years, numerous papers have been published regarding autonomic changes in GTCS and detection of this subtype of seizure (6); those findings have given hope to people with epilepsy (PwE) suffering from GTCS that quicker interventions during seizures and better prevention of SUDEP is possible, even though no quality evidence is present to support the claim that increased knowledge, detection and diagnosis of GTCS have led to decreased SUDEP mortality (7). To the contrary, little research has been conducted regarding peri-ictal changes in focal with impaired awareness seizures (FIAS) and their link to SUDEP. In this work, we retrospectively analyzed the interictal (awake and sleep) and peri-ictal heart rate variability (HRV), as well as peri-ictal heart rate (HR) and breathing rate (BR) of FIAS of a patient who suffered from a “probable SUDEP” 3 weeks following presurgical investigation at our epilepsy monitoring unit. The goal was to determine whether autonomic changes could be seen in between or during FIAS, and assess whether these changes, if seen, could be considered as potential markers for an increased risk of SUDEP.

## 2. Methods

This study was performed in accordance with the guidelines set forth by our local Institutional Review Board regarding patient consent. Written consent was obtained from next of kin.

### 2.1. Patients

The SUDEP patient’s medical chart was reviewed to retrieve clinical information, neuroimaging findings, and video-electroencephalography (VEEG) results. We also included a control group of two living male patients from our local database matched for age, sex, and location of the epileptic focus (confirmed by continuous video-surface EEG).

### 2.2. Interictal and peri-ictal HRV analyses

Electrocardiogram (ECG) recordings of the SUDEP patient and the living control group, acquired during VEEG monitoring, were used to retrospectively analyze HRV. In order to study *interictal* HRV, six 5-min awake segments and six 5-min sleep stage II (N2) segments were selected following recommendations by Myers et al. (8) for HRV analysis in patients with epilepsy: (1) at least 8 h after the last tonic–clonic seizure; (2) at least 1 h after the last known electroencephalographic seizure, and (3) at least 1 h before the subsequent seizure. For *peri-ictal* HRV analysis, electroclinical seizures which lasted more than 2 min with a reliable R peak detection on ECG recordings were chosen; the minimal 2 min duration established here is often used for ultra-short term HRV analysis and represents the minimal window for root mean square of successive RR interval differences (RMSSD) analysis in athletes (9). The preictal and postictal periods were, respectively, 6 and 18 min in duration, each divided into three and nine 2-min epochs (18 min were used here vs. 17 min for the cardiorespiratory analysis described below to allow us to have nine epochs of equal duration, 2 min in this case). The interictal and peri-ictal ECG data were exported from the VEEG recording system (Nihon Koden) into European data format (EDF) files. The Brain Vision Analyzer 2.1 commercial software was initially used to identify R peaks from ECG data, then visually inspected to detect and manually correct artifacts, missed beats, or ectopic beats. Ectopic beats were removed from the recordings and replaced by an interpolated R-R interval. The Kubios HRV 3.2.0 software was used to calculate the standard HRV parameters, including the RMSSD, low-frequency power (LF), high-frequency power (HF), and the LF/HF ratio (10). The Wilcoxon Signed-Rank statistical test was used to assess whether a statistically significant difference of HRV metrics existed between the patient with SUDEP and the controls. Bonferroni correction was applied to account for multiple comparisons. The significance level (alpha) using this correction was set at α = 0.0125.

### 2.3. Cardiorespiratory analysis

A postictal cardiorespiratory analysis was conducted on selected seizures of the SUDEP patient and of the control group using the BR and HR as measured with respiratory bands embedded within the Hexoskin smartwear (Carré Technologies Inc., Montreal, Canada), which was worn by the patients during their stay at the epilepsy monitoring unit. Inclusion criteria for analyzed seizures were the following: (1) FIAS that lasted more than 45 s, (2) without evolution to bilateral convulsive seizures. These inclusion criteria, applied to the active control group, were used to ensure that seizures of similar duration and type (FIAS) to those of the SUDEP patient were used; minimum seizure duration for the SUDEP patient was 58 s. The postictal period was defined as the 17-min period after seizure termination. Postictal duration was based on the MORTEMUS study, where SUDEP was seen up to 17 min postictally (4). Apnea and hypopnea were defined as a complete absence of respiratory movements for ≥10 s and as a reduction of ≥50% in ventilation with signs of arousal, respectively, based on guidelines from the 2017 American Academy of Sleep Medecine Manual (11).

It is important to note that for the analyses that were performed here, we did not consider that the control group had “0 risk of SUDEP.” As they are still alive at this moment, SUDEP risk will be present until they suffer from a non-SUDEP death. However, we based our analysis on the principle that the controls, when compared to the SUDEP case, were less at risk of SUDEP at the same age range because they were still alive. This allowed us to infer that findings in the control group could reasonably be compared with the SUDEP case and suggest hypotheses based on those findings.

## 3. Results

### 3.1. Case presentation

A 33-year-old male, with drug-resistant epilepsy since age 22 years, was admitted to our epilepsy monitoring unit for presurgical assessment. He reported having 2–3 FIAS per month without or with an aura (visual flashes, dizziness). Focal to bilateral tonic–clonic seizures (FBTCS) occurred once per year on average, with two of them evolving into status epilepticus in the context of nonadherence to anti-seizure medication (ASM). His cardiac history was unremarkable apart from a transient increase in the length of the ECG P wave which was observed at the emergency department after two episodes of FBTCS in 2011 and 2016. This anomaly spontaneously resolved on the third-day control ECG for both episodes. Other ECGs were normal.

Upon his admission to our unit, the neurological examination was normal. Neuropsychological evaluation showed significant impairment of both verbal and non-verbal memories. VEEG recordings revealed bilateral temporal interictal epileptiform discharges with left predominance. Twelve FIAS (eight during sleep and four while awake) and two electrical seizures (both while awake) were recorded, with a left temporal onset in eight and right temporal in three; three were non-lateralized. One seizure occurred prior to medication withdrawal (brivaracetam 200 mg/day, extended-release carbamazepine 1,200 mg/day and clobazam 20 mg/day), one after brivaracetam was weaned, 10 after both brivaracetam and carbamazepine had been weaned, and two while off all antiseizure medications. No postictal generalized EEG suppression patterns were observed. Brain MRI identified left mesial temporal sclerosis and a small right anterior temporal cavernous angioma. Positron emission tomography scan revealed left anterior temporal hypometabolism. Ictal single emission computed tomography showed an activation in the left anterior temporal lobe which extended to the inferior insula. At day 12, the patient was discharged with lacosamide (200 mg/day), extended-release carbamazepine (1,200 mg/day), and clobazam (10 mg/day). Lacosamide was only started upon discharge. At the 2-week follow up, the patient reported no seizures. Three weeks after his discharge, the patient died during the night, at home, alone. A coroner’s investigation, without autopsy, concluded that the most probable cause of death was an epileptic seizure. His death fulfilled the criteria for a “probable SUDEP” (1).

### 3.2. Presentation of controls

The two control patients were aged 34 and 24 years old, respectively. Both lived with their family and were not married; it is uncertain whether they shared a room with a family member. Duration of epilepsy was 20 and 6 years, respectively. Both controls had around 3–5 FIAS seizures per month, with a history of FBTCS in the last year. Both have had nocturnal seizures (albeit not exclusively) though none were recorded during their stay in the EMU. The first control patient had a congenital infarct in the right middle cerebral artery territory. The second control patient had non-lesional bitemporal lobe epilepsy confirmed by VEEG recordings. The first control patient was taking levetiracetam (4 g/day), valproic acid (1,250 mg/day), and topiramate (300 mg/day) while the second was on eslicarbazepine (1,000 mg/day), topiramate (200 mg/day), and clobazam (30 mg/day).

### 3.3. Interictal HRV

Table 1 shows awake and sleep HRV values of the SUDEP patient compared to control subjects with bitemporal lobe epilepsy (biTLE) matched for age and sex. All awake HRV parameters except LF/HF values were significantly lower for the SUDEP patient as compared to those of the control subjects (*p* < 0.0125). Similarly, during N2 stage sleep, all parameter values of the SUDEP patient except LF were significantly lower than those of the control subjects (*p* < 0.0125 for RMSSD, HF, and LF/HF).

**Table 1:** Awake and sleep HRV analysis results.

HRV metrics	Awake state		
	SUDEP Patient—Median (IQR)	Non-SUDEP biTLE patients (2)—Median (IQR)	Value of *p*
RMSSD (ms)	33.3 (27.0–36.3)	66.0 (44.7–78.5)	0.0027^*^
LF (ms^2^)	942.9 (601.4–1318.4)	2961.7 (1867.0–1983.0)	0.0014^*^
HF (ms^2^)	215.3 (152.0–254.2)	1187.7(451.1–2500.4)	0.0011^*^
LF/HF	5.1 (2.7–6.9)	3.3 (2.3–4.9)	0.1252
	**Sleep state (N2 sleep stage)**		
	**SUDEP patient - Median (IQR)**	**Non-SUDEP biTLE patients (2) - Median (IQR)**	**Value of *p***
RMSSD (ms)	28.9 (26.4–31.8)	85.8 (68.9–99.9)	*0*.0003*
LF (ms^2^)	918.4 (506.4–1462.8)	1709.1 (1086.5–3,168)0.0	0.0329
HF (ms^2^)	255.4 (106.3–269.5)	2161.2 (1555.8–2897.6)	0.0003*
LF/HF	6.6 (2.3–11.9)	0.98 (0.5–1.8)	0.0013*

biTLE, bitemporal lobe epilepsy; HRV, heart rate variability; RMSSD, root mean square standard deviation; HF, high frequency; LF, low frequency; REM, rapid eye movement; IQR, interquartile range; SD, standard deviation; ^*^statistically significant, after Bonferroni correction.

### 3.4. Peri-ictal HRV

**Figure 1:**
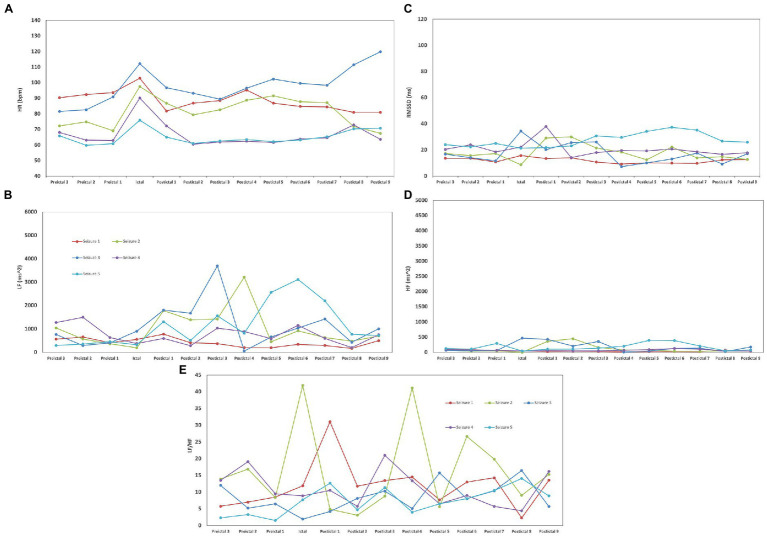
Heart rate variability (HRV) changes during preictal, ictal, and postical periods of five focal with impaired awareness seizures (FIAS) for the sudden unexpected death in epilepsy (SUDEP) patient. **(A)** For all seizures of both groups, we observed the same pattern of an increase in HR during the seizure and a decrease in postical HR when compared to the preictal state. In seizures 2 and 3, postictal mean HR was increased when compared to preictal mean HR (19.05–20.17%). **(B)** Delayed LF postical recuperation was seen, particulary in the nocturnal seizure of the SUDEP patient. In all seizures of the SUDEP patient, LF continued to increase in the first postictal period (39.8–819%) when compared to ictal LF. **(C)** In the SUDEP patient, a 21.97% increase in ictal RMSSD was seen when compared to preictal median RMSSD. Preictal RMSSD were 59.92–75.24% lower than those of the controls, with a statistically significant difference being noted in preictal RMSSD values between the two groups (*p* < 0.05). **(D)** HF values were 92.45–97.18% lower than those of the controls. **(E)** LF/HF ratio was increased in all epochs in the SUDEP case when compared to controls; there was a significant difference in the postictal LF/HF ratio between the SUDEP patient and controls (*p* < 0.05). In seizure 1 of the SUDEP patient, a pattern of important increase (161.24%) and decrease (62.05%) was seen between the ictal period and second postictal period. In seizure 2 of the SUDEP patient, a similar pattern was observed, with LF/HF ratio being increased by 401.40% during the seizure before decreasing by 88.31% during the 1^st^ postictal epoch. A similar increase in LF/HF (368.33%) and decrease (86.33%) was seen between the third and fifth postictal periods. No similar variations were noted in the other three seizures of the SUDEP patient.

**Figure 2:**
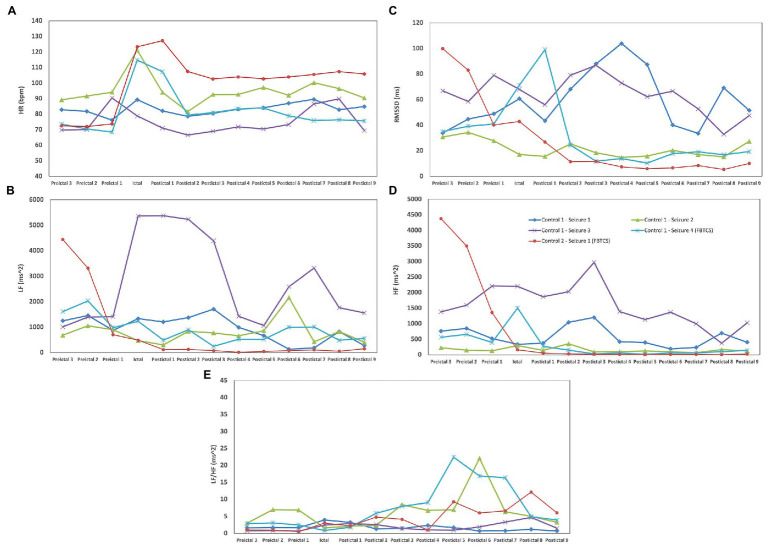
HRV changes during preictal, ictal, and postical periods in five seizures of two controls with biTLE. **(A)** For all seizures of both groups, we observed the same pattern of an increase in HR during the seizure and a decrease in postical HR when compared to the preictal state. **(B)** In 4/5 seizures of the control group, postictal LF recuperation was seen immediately after seizure termination; in 1/5 seizures of the control group (seizure 3 of control #1), LF postictal recuperation was only seen 8 min following seizure termination. **(C)** In controls, RMSSD generally increased during seizures, followed by an important decrease in postictal. **(D)** No postictal mean HF increase was noted in both controls when compared to preictal HF. **(E)** In seizure 2 of control #1, an important and rapid increase (218.33%) and decrease (71.07%) in LF/HF was seen between the fifth and seventh postictal periods. No similar variations were noted in the other four seizures of the controls.

For peri-ictal HRV analyses, the SUDEP patient had five FIAS that met our inclusion criteria (duration of >2 min and with clearly identifiable R peaks), of which, four were diurnal (seizures 1, 3–5), and one was nocturnal (seizure 2). Five seizures of the control group were also selected to analyze peri-ictal HRV (three FIAS and two FBTCS). None of the control patients had nocturnal seizures during their VEEG. In addition to FIAS, two FBTCS were selected from controls to compare changes seen in FIAS of the SUDEP patient to FBTCS from controls. This allowed us to determine whether FIAS of the SUDEP patient were more severe from an autonomic standpoint, and therefore evaluate if FIAS could be used as reliable markers of SUDEP risk, similarly to FBTCS in the current literature. Figure 1 shows HRV changes during preictal, ictal, and postical periods of the five FIAS from the SUDEP patient. Figure 2 shows HRV changes during preictal, ictal, and postical periods of the five seizures of the two living control subjects. Table 2 shows pre-ictal, ictal, and post-ictal results for the HRV analysis comparing the SUDEP case and controls.

**Table 2:** Peri-ictal HRV analysis results.

HRV metrics	SUDEP Patient—Median (IQR; n_seizures = 5)	Non-SUDEP biTLE patients (2)—Median (IQR) (n_seizures = 5)	*p* value
Pre-Ictal			
HR (bpm)	73.3 (72.6–75.6)	78.0 (76.7–81.5)	0.8337
RMSSD (ms)	17.5 (16.4–18.2)	43.6 (40.5–45.9)	0.0121^*^
LF (ms^2^)	568.6 (478.1–696.0)	1262.0 (1118.0–1355.6)	0.0366
HF (ms^2^)	76.5 (67.6–92.1)	566.9 (503.0–648.2)	0.0121^*^
LF/HF	7.2 (6.2–8.7)	2.2 (2.0–2.4)	0.0601
Ictal			
HR (bpm)	97.5 (90.2–102.9)	114.8 (89.3–121.1)	0.4009
RMSSD (ms)	21.4 (15.8–22.2)	60.7 (42.9–68.0)	0.0601
LF (ms^2^)	378.7 (328.9–557.3)	1234.7 (486.4–1333.5)	0.0949
HF (ms^2^)	42.6 (42.5996–46.9023)	337.1 (297.3–1506.2)	0.0601
LF/HF	8.9 (7.7–11.9)	2.4 (1.6–3.0)	0.0601
Post-Ictal			
HR (bpm)	78.2 (75.4–80.1)	81.2 (79.6–84.1)	0.6745
RMSSD (ms)	18.0 (14.6–20.8)	33.6 (26.5–39.8)	1.0000
LF (ms^2^)	761.0 (521.0–1140.5)	965.6 (546.9–1414.0)	0.6745
HF (ms^2^)	73.8 (47.7–117.2)	254.9 (215.9–404.0)	0.6745
LF/HF	9.5 (6.8–13.5)	3.4 (2.1–4.9)	0.0120*

biTLE, bitemporal lobe epilepsy; HRV, heart rate variability; RMSSD, root mean square standard deviation; HF, high frequency; LF, low frequency; REM, rapid eye movement; IQR, interquartile range; SD, standard deviation; ^*^statistically significant, after Bonferroni correction.

#### 3.4.1. Heart rate

For all seizures of both groups, we observed the same pattern of an ictal increase in HR followed by a decrease in the postictal period. HR decreased after seizure termination in both the SUDEP patient and controls, but mean postictal HR was higher than preictal mean HR in both groups (Table 2).

#### 3.4.2. Root mean square of successive differences between normal heartbeats

The SUDEP patient had preictal RMSSD values that were 59.92–75.24% lower than those of the controls (Table 2), with a statistically significant difference being noted in preictal RMSSD values between the two groups (*p* < 0.05). In the SUDEP patient, a 21.97% increase in ictal RMSSD was seen when compared to preictal median RMSSD. In controls, RMSSD increased during seizures (22.37%), followed by an important decrease when compared to the median preictal value (−137.45%).

#### 3.4.3. Low frequency

Preictal LF values were higher in controls than in the SUDEP case but these differences were not statistically significant after Bonferroni correction (*p* > 0.0125). Also, in all seizures of the SUDEP patient, LF postictal recuperation was not seen immediately in the first postictal epoch, particulary for the nocturnal seizure; LF continued to increase in the first postictal period (39.8–819%) when compared to ictal LF (Figure 1). On the other hand, in all seizures of both controls, LF postictal recuperation was seen immediately after the first postictal epoch, with no continued increase in LF following seizures (Figure 2).

#### 3.4.4. High frequency

The SUDEP patient had preictal HF values that were 92.45–97.18% lower than those of the controls, with this difference being statistically significant (*p* < 0.05). In 2/5 seizures (seizures 2 and 3 of Figure 1) of the SUDEP patient, postictal HF was increased when compared to preictal HF (75.98–120.92%), but mean postictal HF of all combined seizures was not increased when compared to preictal HF. In both controls, the postictal mean HF was not increased when compared to preictal HF.

#### 3.4.5. LF/HF ratio

LF/HF ratio was increased in all epochs of the SUDEP case when compared to controls; there was a significant difference in the postictal LF/HF ratio between the SUDEP patient and controls (*p* < 0.05). In seizure 1 of the SUDEP patient, an initial pattern of important increase (161.24%) followed by decrease (62.05%) in LF/HF Ratio was seen between the ictal period and second postictal period. In seizure 2 of the SUDEP patient, a similar pattern was observed, with LF/HF ratio being increased by 401.40% during the seizure before decreasing by 88.31% during the first postictal epoch. A similar increase in LF/HF (368.33%) and decrease (86.33%) was seen between the third and fifth postictal periods. No similar variations were noted in the other three seizures of the SUDEP patient. In seizures of both controls, a similar pattern was only seen in seizure 2 of control subject #1 (see Figure 2), but with a late onset, between the fifth and seventh postictal epochs. All patterns mentioned above can be seen in Figures 1, 2.

### 3.5. Cardiorespiratory analysis

**Figure 3:**
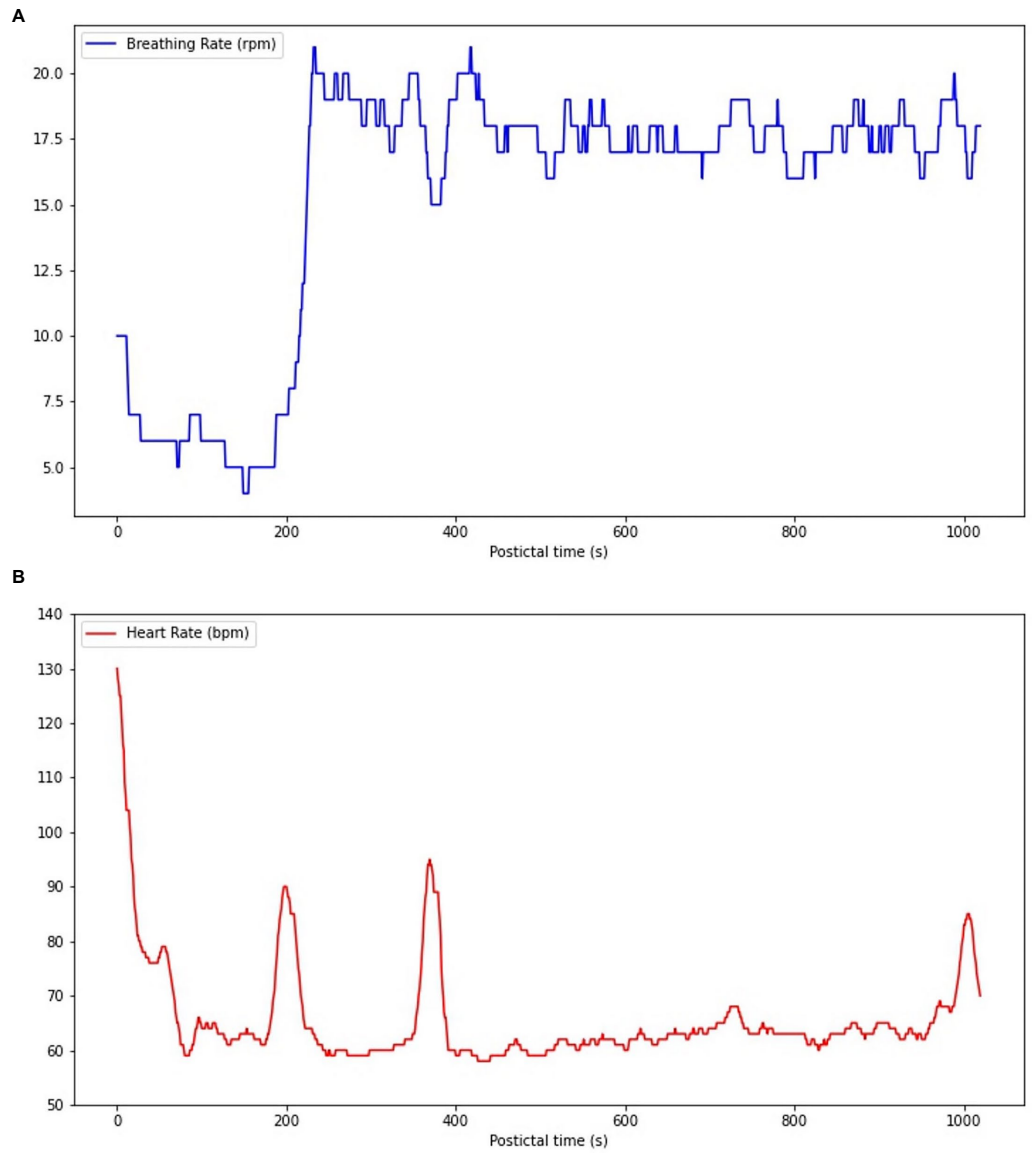
Cardiorespiratory functions during the postictal phase following one of the SUDEP patient’s FIAS. **(A)** A hypopnea (mean BR = 9.9 rpm) immediately follows seizure termination for 12 s, with BR decreasing to 4 rpm. **(B)** A tachycardia (mean HR = 116 bpm) is present following seizure termination for 15 s, but HR rapidly decreases to 59 bpm, resulting in a bradycardia for 10s (mean HR = 59.4 bpm).

For the cardiorespiratory analysis, 14 seizures of the SUDEP patient and five seizures of the control group matched our inclusion criteria. Of those seizures, 13/14 of the SUDEP patient’s seizures and 4/5 of the control group’s seizures were unremarkable (no abnormal cardiorespiratory patterns seen during the postictal epochs) and thus are not described here. Figure 3 illustrates the delayed postictal cardiorespiratory recuperation observed following one of the SUDEP patient’s seizures. The FIAS (lasting 90 s) was followed by immediate and simultaneous tachycardia (mean HR = 116 bpm) and hypopnea (mean BR = 9.9 rpm) for 15 and 12 s, respectively. This was immediately followed by an abrupt decrease in HR resulting in bradycardia for 10 s (mean HR = 59.4 bpm) and more severe hypopnea for 202 s (mean BR = 6.4 rpm); in comparison, the patient’s HR and BR at rest were 74 bpm and 15 rpm, respectively. In one of our control’s seizures, a similar pattern of simultaneous hypopnea and bradycardia was observed, with BR and HR between 10–6 rpm and 60–58 bpm, respectively, for 8 s (between 279 and 288 s postictally). However, the onset of this abnormal pattern was seen more than 4 min following seizure termination (Figure 3).

## 4. Discussion

### 4.1. SUDEP risk factors

The SUDEP patient had three main risk factors for SUDEP, namely a history of GTCS, the presence of nocturnal seizures and the fact that he was sleeping alone. A recent study showed that the risk of SUDEP increased 15- and 5-fold in patients with nocturnal GTCS in the last year of observation and in patients living alone, respectively (5). Other SUDEP risk factors present in this patient were the following: refractory epilepsy, temporal lobe epilepsy, and a history of GTCS (5). Temporal lobe epilepsy has been reported to increase the severity of postictal hypoxemia when compared to extratemporal lobe epilepsy (12). It has also been shown to induce abnormal and sustained brainstem and autonomic activation when compared to frontal lobe epilepsy, mainly in the postictal phase (13), potentially leading to parasympathetic hyperactivity, and thus increased SUDEP risk (14). Furthermore, this patient had biTLE, with a left temporal onset predominance (eight left temporal vs. three right temporal). In a recent study, left temporal lobe epilepsy (L-TLE), when compared to right temporal lobe epilepsy (R-TLE) showed increased parasympathetic activity (15). L-TLE predominance therefore could have put the patient at an increased risk of elevated peri-ictal parasympathetic tone, and thus SUDEP. Furthermore, presence of R-TLE also suggests a potential risk of sympathetic overdrive, which can lead to autonomic disturbances and imbalances when coupled with L-TLE. Ultimately, biTLE leads to risk of autonomic disturbances related to important variations in sympathetic and parasympathetic activity.

### 4.2. Awake and sleep HRV findings

During resting awake time and N2 sleep stage, RMSSD, LF, and HF were all reduced in the SUDEP patient when compared to age and sex-matched controls with biTLE. Reduced short-term LF is currently considered as a valid biomarker for sudden cardiac death (16) and was associated with SUDEP in a recent study on 31 SUDEP cases and 56 living epileptic controls (17), while increased HF at rest was associated with longer survival and was potentially cardioprotective in SUDEP in this same study (17). HF and RMSSD reflect parasympathetic activity to the heart (vagal drive) (18), and an increase in vagal tone was found to be cardioprotective (19). This is further supported by the fact that a 25% reduction of SUDEP events was seen in patients with refractory epilepsy who were treated with adjunctive vagus nerve stimulation (VNS) therapy when compared to patients with refractory epilepsy not treated by VNS (20). However, while vagal tone can be considered as cardioprotective, SUDEP cases in VNS patients have been reported; while no hypothesis has been proposed to why VNS therapy does not eliminate SUDEP, this is most probably due to the inability of VNS to prevent complete recurrence of FBTCS, the main SUDEP risk factor (5). Also, many deaths resulting from SUDEP are due to pillow suffocation, as this is purely mechanical and not related to autonomic and physiological functions (18).

Increased RMSSD at rest was seen in high level athletes during high training loads in preparation for competitions (19). Increased awake and sleep RMSSD and increased sleep LF were seen in moderately trained individuals when compared to sedentary individuals (20). Lower HF, LF, and RMSSD, as seen in our patient, were seen in patients with chronic refractory epilepsy when compared to healthy controls; in the same study, RMSSD was negatively correlated with SUDEP-7 risk (21). This suggests that people with chronic refractory epilepsy, such as the SUDEP patient, do not have the cardiovascular adaptations to training and efforts, from frequent seizures in this case, seen in moderately and highly trained athletes. Those cardiovascular adaptations would protect the patient from a potential cardiopulmonary breakdown resulting from the consequences of intensive and sustained ictal manifestations of a FBTCS that would lead to important oxygen demand, and thus desaturation. This non-adaptative cardiovascular state could have put this patient at risk in the context of an intense and brief physical and cerebral effort, as seen in a FBTCS. Therefore, the findings mentionned above are in line with our results, which suggest that the SUDEP patient, with reduced HF, RMSSD, and LF at rest, could potentially have been at an increased risk of SUDEP; he might not have had the expected cardioprotective adaptation to his epilepsy. Based on this analysis, this patient could have had autonomic dysfunctions, possibly due to abnormalities in the brainstem’s cardiorespiratory regulation centers. The transient anomalies of the length of P-waves observed after two episodes of FBTCS (see *Case presentation*) possibly reflect the increased cardiorespiratory vulnerability in this patient.

### 4.3. Peri-ictal HRV findings

**Figure 4:**
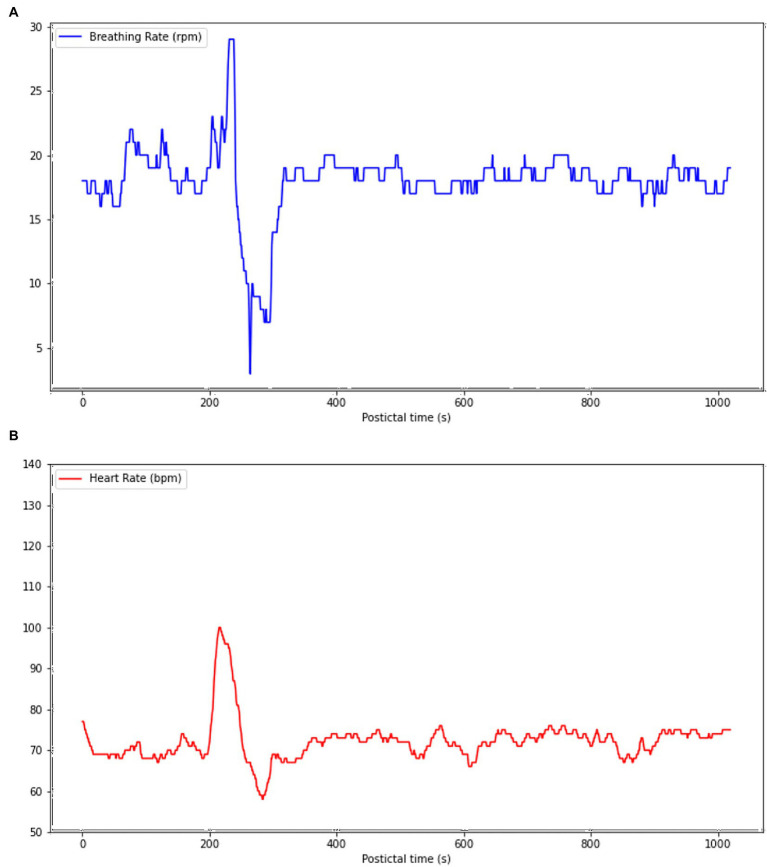
Cardiorespiratory functions during the postictal phase following one control’s focal seizure with awareness (FAS). **(A)** Hyperpnea is present 200 s following seizure termination, but BR rapidly decreases to 6 rpm, resulting in hypopnea 279 s following seizure termination. **(B)** A tachycardia is present simultaneously to hyperpnea, but HR rapidly decreases simultaneously to BR to a rhythm of 58 bpm 279 s, resulting in abnormal and simultaneous bradycardia and hypopnea.

In two seizures (seizures 2 and 3) of the SUDEP patient, postictal mean HF and HR were increased when compared to preictal mean HF and HR. Increased postictal mean HR (vs. preictal HR) and reduced preictal mean HF (vs. postictal HF) in GTCS are both associated with increased GTCS severity (22). In our SUDEP patient, this pattern was seen with FIASs and not GTCSs, suggesting that the SUDEP patient’s seizures were of an important severity. One could hypothesize that if those patterns are seen in FIAS, which result in less oxygen and energy consumption when compared to FBTCS, any FBTCS in this patient would be this much more severe. In all seizures of the SUDEP patient, LF continued to increase in the first postictal period when compared to ictal LF, while this was not seen in both controls. LF is believed to represent cardiac sympathetic drive to the heart (23). Our patient had an overall increased sympathetic activity seen through all epochs, with an LF/HF ratio that was significantly increased postictally when compared to controls (*p* < 0.05). The sudden sympathetic activity increase following a seizure and the overall increased sympathetic activity through all epochs could suggest that an autonomic shift toward an abnormally increased sympathetic drive, particularly in the postictal period, is occurring which could lead to cardiorespiratory breakdown by increased and sustained workload and oxygen demand, and thus severe dyshomestoasis, and ultimately SUDEP. However, sympathetic activity could also counteract parasympathetic activity, which inhibits cardiorespiratory functions, therefore acting as a potential protective mechanism against depressed vital functions, and thus SUDEP. In two seizures of the SUDEP patient (one nocturnal and one awake), an important increase in LF/HF ratio (368.33–401.40%) was seen followed by an important decrease (86–33-88.31%) after 4 min. LF/HF ratio is believed to represent the autonomic balance between parasympathetic (LF and HF) and sympathetic (LF) activity (23). A rapid increase in LF/HF ratio would suggest an increased and hyperactive sympathetic system while a rapid decrease in LF/HF ratio would suggest an increased and hyperactive parasympathetic activity. The pattern seen in two seizures of our patient, a rapid sympathetic activity increase followed by rapid parasympathetic activity increase, suggests an autonomic vulnerability and hypersensitivity that could lead to severe dyshomeostasis and cardiorespiratory imbalance. The protective effect of sympathetic activity discussed above is therefore less valuable when it precedes parasympathetic activity, as the latter is not countered; cardiorespiratory functions are therefore inhibited after being greatly stimulated, leading to severe imbalance. Variability, especially in LF/HF ratio, can be seen between the five analyzed seizures of the SUDEP patient. Three of the five seizures had a left temporal onset with contralateral spread, one had an unknown onset with bilateral implication and the last one had a right temporal onset with contralateral spread. Therefore, the onset foci are not consistent throughout those seizures, which could explain the difference in HRV parameter variations. As mentionned above, R-TLE and L-TLE have different effects on the autonomic system, with L-TLE showing increased sympathetic activity (15) (Figure 4).

Ultimately, those perictal HRV findings suggest that the SUDEP patient’s FIAS were autonomically more severe and intense when compared to those of other patients with epilepsy. This increased risk of autonomic imbalance might have put him at a higher risk of adverse postictal events. We presume that a more intense seizure, such as a FBTCS occurring in sleep, could have triggerred a fatal autonomic deterioration which led to his SUDEP.

On the other hand, the SUDEP patient had significantly lower preictal RMSSD and HF values when compared to both controls. In a study by Jepessen et al. (2014), a 25-year-old patient who died of SUDEP had increased preictal parasympathetic activity preceding his GTCS when compared to 11 GTCS from 11 male patients with epilepsy with a median age of 30.5 years (24). Although our study yielded opposing results, other HRV parameters seen in our patient and mentioned above suggest that the SUDEP patient presented autonomic dysfunctions frequently encountered in other cases of SUDEP. Therefore, the peri-ictal HRV pattern seen in our patient reinforces the idea that no clear variation in HRV parameters can be identified as a general biomarker of increased SUDEP risk in patients with epilepsy. However, the general tendency in our analyses suggests that this patient had abnormal HRV patterns, which further supports our suggestion that he was at an increased risk of SUDEP.

### 4.4. Postictal cardiorespiratory findings

Following symptomatic seizures, tachypnea and tachycardia are usually observed as adaptative transient cardiorespiratory hyperactivity to enable a fast recovery from the ictal oxygen debt (25). Even in a suboptimal adaptation scenario, any postictal decrease of HR should be compensated by a larger increase of BR, and vice versa. However, one of our patient’s FIAS was followed by severe hypopnea and an abrupt HR decrease shortly after seizure termination (Figure 2). Such an unexpected cardiorespiratory pattern carries a risk of severe and potentially fatal postictal acid–base imbalance (26). This postictal phase resembles, to a lesser extent, the pattern of a neurovegetative breakdown that is known to classically lead to SUDEP, which consists of an initial tachypnea followed by apnea and bradycardia and ending with terminal apnea and asystole (4). The paradoxical cardiorespiratory postictal pattern observed may result from postictal dysfunction of respiratory and cardiovascular centers of the brain stem (27). A study by Park et al. revealed that the cardiac arrythmogenic threshold is lowered when oxygen saturation decreases <90%, therefore suggesting that important ictal and postictal hypoxemia could facilitate the onset of fatal arrythmias such as ventricular tachycardia and sinus arrest (28). Following a FBTCS, one could hypothesize that such an abnormal and disorganized cardiorespiratory pattern can lead to severe dyshomeostasis or SUDEP. A similar pattern was observed in one of our control’s seizures (see Figure 3), but onset of the abnormal pattern was seen more than 4 min following seizure termination while it was seen immediatly after seizure offset in reported SUDEP cases, making it is less likely that those cardiorespiratory changes were directly caused by seizure dyshomeoastasis.

### 4.5. Effects of medication on SUDEP risk

While in general, patients with epilepsy taking ASMs have a reduced SUDEP risk compared to non-treated patients (5), we recognize that some ASMs have potential cardiorespiratory effects. For example, patients with epilepsy on carbamazepine had reduced parasympathetic activity compared to other ASMs, which may pose a risk of SUDEP (29). In a cohort study, 10,241 patients with epilepsy under enzyme-inducing and non-enzyme-inducing ASM were found to be at an increased risk of cardiovascular events when compared to non-epileptic controls (32). Furthermore, in a recent study on transverse brainstem slices from mice, carbamazepine and lamotrigine were found to impair the gasping response during hypoxia, but not during normoxia, suggesting a possible role of those specific sodium channel blocker ASMs in worsening postictal hypoxia (33), and therefore probably increasing SUDEP risk.

### 4.6. Limitations

Our case report has four main limitations: (1) sudden death occurred a week after his epilepsy monitoring unit stay and not during it, hence the lack of data during the dramatic event (31); (2) the patient did not have a FBTCS during VEEG monitoring, thus the lack of data during a convulsive seizure. Any of those elements would have provided a better understanding of autonomic disorders associated with more severe seizures; (3) the small size of this study increases the risk of sampling bias. We performed a selection based on patient availability in our database (seizures recorded during their stay in the EMU) and resemblance to the case (age, sex, and bTLE). Therefore, the selected patients might not represent the general epileptic population; (4) the control patients did not have nocturnal seizures during their stay in the EMU. Thus, we were not able to compare the nocturnal seizures from the SUDEP patient and the controls, which limits the extent of our conclusions. Finally, it should be noted that, for the cardiorespiratory analysis, HR and BR signals were acquired using a wearable device (Hexoskin) instead of regular medical devices (plethysmography and ECG). The Hexoskin was used because it allowed us to continuously measure breathing rhythm and heart rhythm in patients. Main advantages were that it allowed for continuous and automatic recording of physiological signals, and it was less obtrusive for patients. The Hexoskin Smartwear has been previously validated for the acquisition of reliable measurements of these signals (30). Also, acquiring experience in the use of such smartwear in the EMU could potentially lead us to eventually promote its use in an outpatient setting, which may provide new data to increase our understanding of SUDEP.

## 5. Conclusion

This case report shows intriguing cardiorespiratory and HRV features recorded at rest and postictally following a FIAS, weeks prior to SUDEP, that are suggestive of autonomic imbalance, and ultimately of increased SUDEP risk. Although the value of our observations is subject to confirmation by larger studies, it opens up new avenues of research regarding biomarkers of SUDEP risk. If our findings are verified by further studies, these features could help clinicians better assess SUDEP risk even with focal with or without impaired awareness seizures not evolving to bilateral tonic–clonic seizures at the epilepsy monitoring unit.

## Data availability statement

The raw data supporting the conclusions of this article will be made available by the authors, without undue reservation.

## Ethics statement

The studies involving human participants were reviewed and approved by Research Ethics Board of the University of Montreal Hospital Research Center. The patients/participants provided their written informed consent to participate in this study. Written informed consent was obtained from the individual(s) for the publication of any potentially identifiable images or data included in this article.

## Author contributions

DN, EB, and MR contributed to conception of the study. MR, EB, and YL organized the database. YL and TT performed the statistical analysis. YL wrote the first draft of the manuscript. YL, EB, DT, TT, and DN wrote the sections of the manuscript. A-AB, DN, and EB read, and approved the submitted version. All authors contributed to the article and approved the submitted version.

## Funding

This work was supported by the Natural Sciences and Engineering Research Council of Canada (NSERC) (grant #: 415079), and the Institute for Data Valorization (IVADO) (grant #: 51628 and 3927033406).

## Conflict of interest

The authors declare that the research was conducted in the absence of any commercial or financial relationships that could be construed as a potential conflict of interest.

## Publisher’s note

All claims expressed in this article are solely those of the authors and do not necessarily represent those of their affiliated organizations, or those of the publisher, the editors and the reviewers. Any product that may be evaluated in this article, or claim that may be made by its manufacturer, is not guaranteed or endorsed by the publisher.
